# Efficacy and safety of FLT3 inhibitors for acute myeloid leukemia: a network meta-analysis

**DOI:** 10.3389/fonc.2026.1831094

**Published:** 2026-05-11

**Authors:** Yaoyao Xu, Jiaming Li, Yao Gao, Gan Huang, Yingjian Zeng

**Affiliations:** 1 School of Clinical Medicine, Jiangxi University of Chinese Medicine, Nanchang, China; 2 Dongming Community Healthcare Center, Pudong NewArea, Shanghai, China; 3 Hematology Department, Affiliated Hospital of Jiangxi University of Traditional Chinese Medicine, Hematology Department, Nanchang, China

**Keywords:** acute myeloid leukemia, FLT3 inhibitors, network meta-analysis, overall survival, response

## Abstract

**Background:**

Along with the more and more clinical application of various FLT3 inhibitors in acute myeloid leukemia (AML), their real clinical benefits still remain a debated topic. Therefore, this study uses a network meta-analysis method to make comparison on the treatment efficacy and safety situation of different FLT3 inhibitors, hence aiming to offer evidence-based supporting materials for the selection work of clinical treatment strategies.

**Methods:**

A systemic search action was carried out inside PubMed, Web of Science, Cochrane Library, and Embase, from the starting time of each database until December 17, 2025, for the aim to find randomized controlled trials of FLT3 inhibitors used for AML treatment. Stata 18.0 and R Studio software were applied to conduct network meta-analysis, hence RevMan 5.4 software was utilized to perform literature quality appraisal and bias risk assessment.

**Results:**

Twenty RCTs with total 6128 acute myeloid leukemia patients are contained. Efficacy comparison outcomes have demonstrated that treatment with FLT3 inhibitors Gilteritinib (OR = 1.75, 95% CI: 1.16–2.66) and Midostaurin (OR = 1.31, 95% CI: 1.07–1.60) can produce significant improvement in patients’ complete remission rate. Therefore, survival analysis has found that Gilteritinib (HR = 0.70, 95% CI: 0.49–0.99) and Quizartinib (HR = 0.73, 95% CI: 0.54–0.98) can significantly prolong patients’ overall survival (OS). Thus, safety evaluation results have shown that, compared with the control group, the experimental group bears significantly higher risk of adverse events including reduced neutrophil count, anemia, elevated alanine aminotransferase, elevated aspartate aminotransferase, fatigue, thrombocytopenia, dyspnea, and neutropenia (*P* < 0.05).

**Conclusions:**

The investigation outcomes of this research demonstrate that FLT3 inhibitors can produce effective prolongation of overall survival (OS) and bring about improvement to complete remission rate (CR) in AML patients, with good safety and tolerability performance; therefore, hence, gilteritinib may show more excellent treatment effectiveness among them.

**Systematic review registration:**

, identifier CRD420251267673.

## Introduction

Acute myeloid leukemia (AML) is a high-degree heterogeneous hematological malignancy that has its origin in hematopoietic stem/progenitor cells (1, 2). It takes clonal proliferation abnormalities, impaired differentiation, and defective apoptosis as core pathological characteristics, thus clinically showing rapid development speed with five-year survival rate lower than 30% (3), hence seriously endangering the life and health of global population. Even though standardized treatment plan that combines chemotherapy and allogeneic hematopoietic stem cell transplantation has gotten continuous improvements, most patients still confront the difficult situation of continuously high relapse rates and restricted long-term survival gains. For AML subtypes which carry key molecular mutations, this situation is especially true; these subtypes display poorer treatment reaction rates and prognosis, thus presenting a core challenge within AML precision therapy field.

Inside the complicated molecule environment of AML, FMS-like tyrosine kinase 3 (FLT3) mutations are identified as a core bad prognostic marker and treatment target, which show high expression in acute myeloid leukemia cells (4). Approximately one-third of newly diagnosed patients can be detected with FLT3 mutations, which promote leukemia cells’ survival and multiplication. These mutations are mainly divided into two big subtypes: internal tandem duplication (ITD) and tyrosine kinase domain (TKD) point mutations (5–7). Through conformation changes, mutant FLT3 receptors realize ligand-independent dimerization, and activate non-stop the downstream STAT5 (8), MAPK, and PI3K/AKT signal pathways. This action not only strengthens leukemic cell proliferation in a significant way and restrains differentiation and maturation, but also lowers cell sensitivity to chemotherapeutic medicines by controlling anti-apoptotic gene expression. Hence, this greatly raises the relapse risk of patients, and has severe influence on the overall survival of acute myeloid leukemia patients (9). Therefore, targeted suppression of the mutated FLT3 signal pathway has become a key strategy for improving such patients’ prognosis, and the development and clinical translation of related inhibitors have become a research hot spot in the AML treatment area.

FLT3 inhibitors are one kind of tyrosine kinase inhibitors and can specially block the signal which is triggered by FLT3 mutations (10). At present, various FLT3 inhibitors are under research for all-round management of FLT3-mutated AML patients, including remission induction, maintenance therapy before and after hematopoietic stem cell transplantation, and rescue therapy after relapse (11). Along with precision medicine’s development, present clinical FLT3 inhibitors mainly include three drug generations. First-generation agents, which take midostaurin and sorafenib as representatives, carry out multi-target inhibition on FLT3, VEGFR, and PKC. Although they are approved for combined treatment in newly diagnosed FLT3-mutated AML, their lower target specificity makes clinical efficacy and tolerability have space for improvement (12, 13). Second-generation inhibitors like gilteritinib obviously raise selectivity and inhibitory activity against FLT3 mutants via structural optimization (14). They show better complete remission rates and survival benefits in relapsed/refractory AML patients. Some of these drugs have got approval for application in post-transplant maintenance therapy situations. Third-generation FLT3 inhibitors are still in clinical trial stage. However, there is obvious heterogeneity among different FLT3 inhibitors regarding action mechanisms, dosing plans, and safety situations. Furthermore, systematic comprehensive studies about FLT3 inhibitors’ role in acute myeloid leukemia treatment and comparative efficacy evaluations among different FLT3 inhibitors are not sufficient. Therefore, this study uses a network meta-analysis that compares multiple randomized controlled trials to assess clinical application of different types of FLT3 inhibitors, so as to provide scientific evidence for guiding clinicians’ prescription decisions.

## Methods

According to the rules of the Preferred Reporting Items for Systematic Reviews and Meta-Analyses (PRISMA), therefore this research has carried out registration at the International Prospective Register of Systematic Reviews (PROSPERO), and its registration number is CRD420251267673.

### Literature search

Since our database was established until December 17, 2025, we have found out all studies published in four databases—PubMed, Web of Science, Embase, and the Cochrane Library—by using the keywords “Leukemia, Myeloid, Acute”, “Acute Myeloid Leukemias”, “Acute Myelogenous Leukemias”, “Midostaurin”, “Sorafenib”, “Sunitinib”, “Lestaurtinib”, “Tandutinib”, “Quizartinib”, “Gilteritinib”, “Crenolanib”, “FLT3”, and other related keywords in the four databases: PubMed, Web of Science, Embase, and the Cochrane Library. Therefore, we did not set restrictions on publication date or language for the search process to guarantee comprehensiveness. Thus, two researchers took part in the search work; Appendix 1 details the specific search strategy.

### Inclusion criteria

Studies meeting the following criteria were included:

1. Subjects must hold a confirmed AML diagnosis in accordance with diagnostic criteria that gain international recognition.
2. All selected studies must be RCTs; there is no limitation set on their blinding condition. The studies have to be formally issued articles or literature materials that provide accessible full texts and original data.
3. For intervention protocols, the experimental group must accept treatment that uses any FLT3 inhibitor, with the concrete administration method being clearly written down in the literature. Therefore, the control group may receive either standard clinical chemotherapy plans or placebo treatment.
4. The literature material must contain at least one pre-defined efficacy or safety end point that is clearly specified in this study. Therefore, CR or OS can be counted as efficacy end points, and hence, adverse event occurrence rates can be regarded as safety end points.

Exclusion criteria.

1. Study categories that fail to satisfy requirements, specifically containing non-randomized controlled trials for instance cohort studies, case-control studies, case reports, reviews, as well as animal experiments.
2. Literatures from which complete original data cannot be acquired, where key information is absent and cannot be supplemented.
3. Research objects fail to satisfy demands, for instance, ambiguous diagnostic standards of AML, inclusion of sufferers with other hematological malignant diseases such as acute lymphoblastic leukemia, or research objects with serious infections or organ function failure.
4. Intervention measures fail to satisfy related demands, for instance, the experimental group does not employ any kind of FLT3 inhibitor, or there exists no explicit comparability between the intervention measures of the control group and the experimental group.
5. Outcome measures which cannot satisfy related demands, specifically: omission of any pre-set efficacy or safety endpoints belonging to this study; definition of outcome measures with ambiguity; or measurement methods lacking consistency that block extraction of valid data.

### Data extraction

Two separate researchers carried out data extraction work, which recorded study features such as authors, publication year, NCT number, sample size, and intervention protocol; patient demographic information included mean age and gender; outcome measures were OS, CR, and adverse events AE. Therefore, disagreements appearing in the review process were solved via mutual discussion and reaching consensus between the two reviewers. Thus, all data related to studies were arranged and written down by using Microsoft Excel 2021 software.

### Quality assessment

Two separated reviewers carried out quality valuation work on the included randomized controlled trials which assess FLT3 inhibitors for acute myeloid leukemia, applying the Cochrane Risk of Bias Tool. Therefore, risk of bias was graded across seven core domains: random sequence generation, allocation concealment, blinding, outcome assessor blinding, incomplete data reporting, selective reporting, and other biases. Hence, disagreements were solved by means of discussion.

### Data analysis

This research first evaluated between-study heterogeneity via the I² statistic; we adopted random-effects model when I² > 50%, and fixed-effects model when I² < 50%. Next, we utilized gemtc package within R software version 4.5.0 to build a consistency model: model <- mtc.model(network, type=“consistency”, n.chain=4, likelihood=“normal”, link=“identity”, linearModel=“random”), with n.adapt=20000, n.iter=50000, thin=1. For constructing non-consistency model, we still used gemtc package in R software version 4.5.0, with modelume <- mtc.model(network, type=“ume”, n.chain=4, likelihood=“normal”, link=“identity”, linearModel=“random”), and iteration parameters are completely same as consistency model. Hence, Deviance Information Criterion was applied to compare model fitting quality and confirm consistency of evidence; DIC difference < 5 signals good consistency. Finally, we calculated area under cumulative ranking curve value to rank efficacy and safety levels of different FLT3 inhibitors. For binary outcomes like complete response rate and adverse events, we employed odds ratios; for survival data, hazard ratios were used, with confidence intervals set at 95%. Whole analysis workflow was finished using Stata and R software. Results contained network plots, cumulative ranking probability plots, ranking tables, and comparison-adjusted funnel plots for each outcome measure, thus guaranteeing findings are reliable and reproducible.

## Results

### Literature screening flowchart

**Figure 1:**
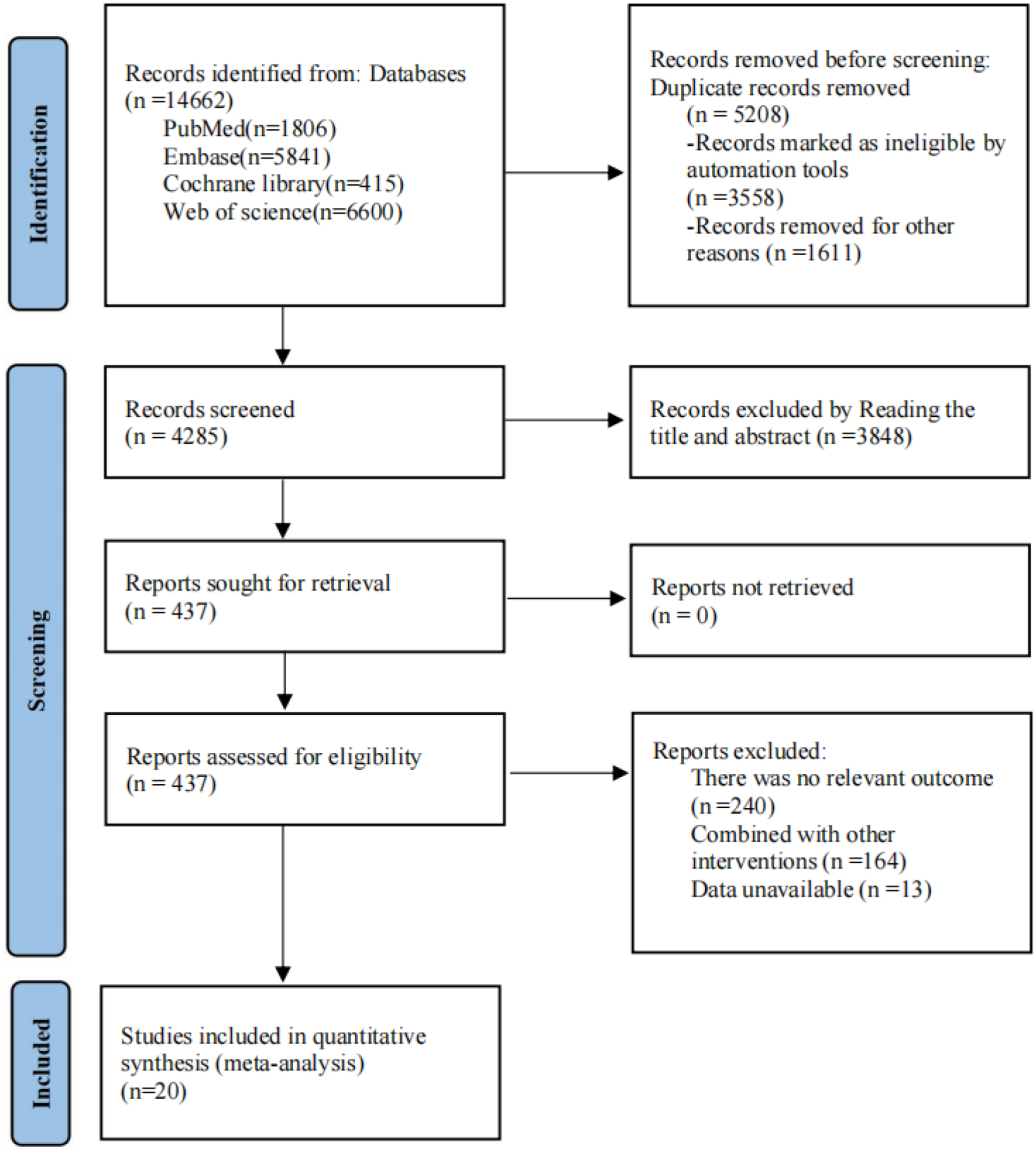
The current study performed a comprehensive literature screening process in strict accordance with the PRISMA guidelines (Preferred Reporting Items for Systematic Reviews and Meta-Analyses). The flow diagram clearly depicts the entire procedure of literature identification, screening, and inclusion used in the present research.

A full-scale information search in PubMed, Web of Science, Cochrane Library, and Embase databases at first found 14,662 related publications. By utilization of EndNote 21 software and manual check work, 5,208 publications were removed from the list. After that, first-step screening of document titles and abstracts, and then full-text examination, excluded those studies which fail to satisfy the inclusion standards. Therefore, finally, 20 publications were selected into the research. **Figure 1** shows and explains the concrete screening process in detail.

### Basic characteristics table

This study has contained 20 RCTs (15–34) that involve 6,128 acute myeloid leukemia patients; among them, 3,328 patients belong to the intervention group and 2,800 patients are in the control group, with 2,966 being male patients and 3,162 being female patients. The interventions under evaluation consist of Gilteritinib (5 studies), Sorafenib (5 studies), Quizartinib (4 studies), Midostaurin (4 studies), and Lestaurtinib (2 studies). Hence, the baseline characteristics of the studies we have included are made into a summary in **Table 1**.

**Table 1:** Characteristics of the included studies at baseline.

Author	Year	Country	NCT	Sample size	Control treatment regimens	Treatment line	Gender (M/F)	Mean age	Intervention	Outcomes
Perl	2019	United States	NCT02421939	Gilteritinib:247 UT:124	MEC, FLAG-IDA, low-dose cytarabine, and azacitidine	Relapsed / Refractory AML, second-line or later therapy	170/201	62 (range 19–85)	Gilteritinib: The dose is 120 mg daily, administered in 28-day cycles, with 1 to 3 cycles of treatment	O1; O2; O3;
Burchert	2020	Germany	DRKS00000591	Sorafenib:43; UT:40	Placebo	post-HCT	41/42	54 (range 18.58–75.58)	Administer 2 tablets (400 mg) daily for 2 weeks (dose level 1), then 3 tablets daily for 4 weeks (dose level 2), followed by maintenance of the full dose of 4 tablets (800 mg) daily for a continuous 24-month	O1; O3;
Röllig	2015	Germany	NCT00893373	Sorafenib: 134; UT:133	Standard chemotherapy (DA induction + HiDAC consolidation) + placebo	Newly diagnosed AML, first-line therapy	134/133	50 (IQR 44–55)	Oral administration: 400 mg twice daily, 400 mg once daily, or 400 mg every other day, for 12 consecutive months.	O1; O3;
Gyan	2025	France	NCT02927262	Gilteritinib: 63; UT:35	Placebo	Newly diagnosed AML, first-line therapy	47/51	64 (range 22–79)	Oral 40mg-120 mg tablets once daily, treatment for up to 2 years or until discon- tinuation criteria were met	O1; O2; O3;
Wang	2022	United States	NCT02752035	Gilteritinib:74; UT:49	Azacitidine	Newly diagnosed AML, first-line therapy	70/53	77 (range 59–90)	120 mg/d orally. a 28- day cycle.	O1; O2; O3;
Erba	2023	Spain	NCT02668653	Quizartinib:268 ; UT: 271	Placebo plus standard chemotherapy (cytarabine + daunorubicin/idarubicin) followed by placebo maintenance	Newly diagnosed AML, first-line therapy	294/245	56 (IQR 46.0–65.0)	Oral administration: 40mg once daily for 3 years	O1; O3;
Serve	2013	Germany	NCT00373373	Sorafenib:102; UT:95	Placebo plus standard chemotherapy (cytarabine, daunorubicin) and placebo maintenance	Newly diagnosed AML, first-line therapy	111/86	68 (range 61–80)	Oral administration 200- 400 mg twice daily for 1 year	O1; O2; O3;
Cortes	2019	United States	NCT02039726	Quizartinib:24; UT:122	Salvage chemotherapy (LDAC, MEC, FLAG-IDA)	Relapsed/refractory FLT3-ITD AML, second-line therapy	177/190	57.5 (IQR 44.0–66.0)	Oral administration: 30– 60 mg once daily, in 28- day cycles	O1; O3;
Wang	2024	China	NCT03182244	Gilteritinib:116; UT:118	Salvage chemotherapy (FLAG, MEC, low-dose cytarabine [LoDAC])	Relapsed/refractory FLT3-mutated AML, second-line therapy	108/126	50.5 (IQR not reported)	Oral administration: 40– 200 mg once daily, in 28- day treatment cycles	O1; O2; O3;
Xuan	2020	China	NCT02474290	Sorafenib: 100; UT:102	Best supportive care (BSC) plus placebo	Newly diagnosed AML (post-HSCT), first-line therapy	102/100	35 (IQR26–42)	Oral administration: 200–400 mg once or twice daily for 180 days	O2; O1; O3;
Voso	2020	Italy	NCT00651261	Midostaur: 83; UT: 80	Placebo plus standard chemotherapy (daunorubicin + cytarabine) + placebo maintenance	Newly diagnosed FLT3-TKD–mutated AML, first-line therapy	79/84	48.8 (range 19.3–59.9)	Received 12 28-day cycles of maintenance therapy, with 50 mg oral midostaurin twice per day daily	O1; O2;
Levis	2024	United States	NCT02997 202	Gilteritinib:178; UT:178	Placebo	Newly diagnosed AML, first-line therapy	183/173	53 (range 18–78)	120 mg per day gilteritinib for 24 months	O1; O3;
Levis	2011	United States	NCT00079482	Lestaurtinib:112; UT:112	Salvage chemotherapy (MEC or HiDAC)	Relapsed/refractory AML, second-line therapy	103/121	56.5 (range 20–81)	Oral administration: 80 mg twice daily for 112 days	O2; O3;
Montesinos	2025	United States	NCT04107727	Quizartinib:180; UT: 93	Placebo plus standard chemotherapy (idarubicin + cytarabine) and placebo maintenance	Newly diagnosed AML, first-line therapy	157/116	57.5 (range 19–70)	60 mg once daily, treatment period ranges from approximately 1 month to a maximum of 20 months	O1; O3;
Larson	2021	United States	NCT01757535	Midostaurin:360; UT: 357	Placebo plus daunorubicin + cytarabine and placebo maintenance	Newly diagnosed AML, first-line therapy	319/398	48 (range 18–61)	50 mg was given twice daily, 28 days per cycle, maintained for 12 cycles	O2; O1;
Schlenk	2025	Germany	NCT02668 653	Quizartinib: 268; UT:271	Placebo plus standard chemotherapy (daunorubicin/idarubicin + cytarabine) and placebo maintenance therapy	Newly diagnosed AML, first-line therapy	245/294	56 (range 20–75)	36 cycles (28 days per cycle) of quizartinib 60 mg daily	O1; O3;
Stone	2017	United States	NCT00651 261	Midostaurin :360 ; UT:357	Placebo plus standard chemotherapy (daunorubicin + cytarabine induction, high-dose cytarabine consolidation) and placebo maintenance therapy	Newly diagnosed AML, first-line therapy	319/398	47.9 (range 18–60.9)	50 mg orally twice daily, for twelve 28-day cycles	O1; O2; O3;
Maziarz	2021	United States	NCT01883362	Midostaurin: 30; UT:30	Standard of care	Newly diagnosed AML, first-line therapy	34/26	52 (range 20–68)	50 mg orally twice daily continuously in twelve 4- week cycles	O1; O3;
Knapper	2017	United Kingdom	ISRCTN17161961; ISRCTN55675535	Lestaurtinib:88; UT:87 Lestaurtinib:212; UT:113	Standard induction (daunorubicin + cytarabine) plus high-dose cytarabine consolidation plus placebo maintenance	Newly diagnosed AML, first-line therapy	77/98 155/170	47 (range 16–66) 50 (range 6–58)	80mg orally twice daily , with a 28-day cycle, for a maximum of 4 cycles	O1; O2;
Loo	2023	Australia	ACTRN12611001112954	Sorafenib :65; UT :33	Idarubicin plus cytarabine induction, cytarabine consolidation, and placebo maintenance	Newly diagnosed AML, first-line therapy	41/57	49.5 (range 18–65)	400 mg orally twice daily, 28-day cycle for 12 consecutive cycles	O1; O2; O3;

This table summarizes key details of the included trials, patient demographic profiles, and the specific dosing regimens for FLT3 inhibitors. M/F, Male/Female.

UT, Usual Therapy; O1, Overall survival(OS); O2, the percentage of patients with complete remission with full or partial hematologic recovery (CR/CRh); O3, Adverse events(AE); Low-dose cytarabine, (LDAC); FLAG-IDA, (G-CSF + fludarabine + cytarabine + idarubicin); MEC, (mitoxantrone + etoposide + cytarabine); HiDAC, (high-dose cytarabine)

### Methodological quality and risk of bias assessment

**Figure 2:**
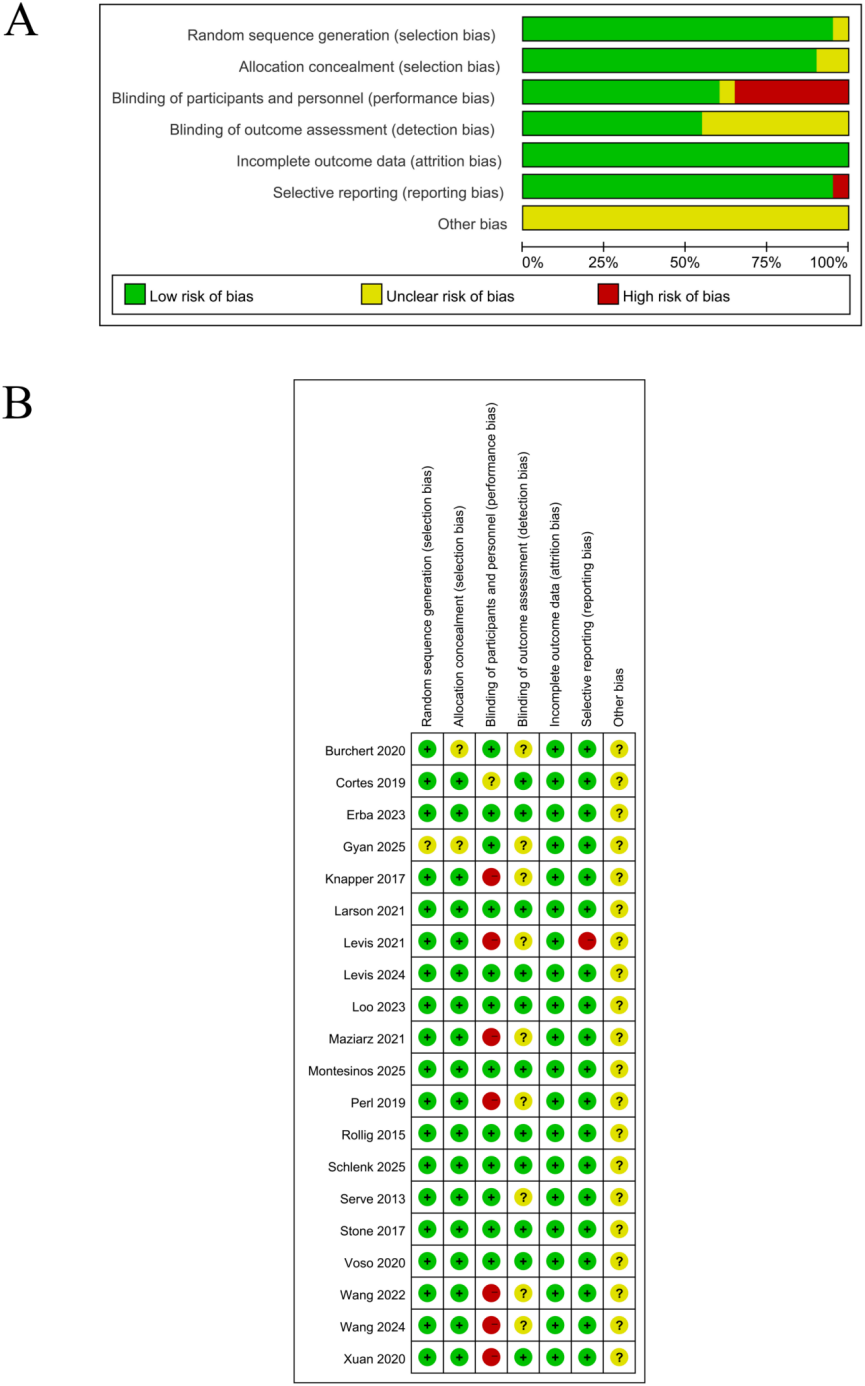
Risk of bias assessment: **(A)** Percentage of risk of bias; **(B)** Summary of risk of bias. **(A)** summarizes the distribution of studies categorized by low, moderate, and high levels of bias risk across each assessed domain; **(B)** meanwhile, presents the detailed, individual-level evaluations of bias risk for each included study.

The methodological quality and risk of bias assessment results are shown in **Figures 2A, B**. All RCTs have detailed seven assessment methods: random sequence generation, allocation concealment, blinding, blinding of outcome assessors, incomplete data, selective reporting, and other biases. Therefore, the cross-study bias risk is divided into low, unclear, or high levels. The majority of studies showed low bias risk in five key fields: random sequence generation, allocation concealment, blinding of outcome assessors, incomplete data, and selective reporting. Furthermore, each study has detailed their protocols and outcomes. Hence, all trials are classified as having unclear bias risk concerning other possible bias sources.

### Paired meta-analysis results

A pairwise meta-analysis concerning CR rates contained four head-to-head comparison works, with their detailed results shown in **Table 2**. Four studies made comparison between standard therapy and Gilteritinib, and heterogeneity test displayed that no significant heterogeneity existed (I² = 0%). Through pooled analysis, we can see that the CR rate in Gilteritinib group is obviously higher than that in control group, with a statistically notable difference (OR = 0.58, 95% CrI: 0.36–0.95, P < 0.05). Three studies compared standard therapy with Lestaurtinib, and they showed low heterogeneity (I² = 19.0%). Pooled results told us that no statistically significant difference appears in CR rates between the two groups (OR = 0.98, 95% CrI: 0.54–1.78, P > 0.05). Three studies carried out comparison between standard therapy and midostaurin, with no heterogeneity (I² = 0%). The CR rate difference between groups has no statistical significance (OR = 0.75, 95% CrI: 0.52–1.08, P > 0.05). Three studies made comparison between conventional therapy and sorafenib, and moderate heterogeneity was exhibited (I² = 49.3%). Pooled analysis showed that no significant difference exists in CR rates between these two groups (OR = 1.35, 95% CrI: 0.81–2.13, P > 0.05).

**Table 2:** Pairwise meta-analysis of complete remission rate for FLT3 inhibitors in AML.

Outcomes	Pairwise meta-analysis	No of study	Heterogeneity (%)	OR 95%CrI
CR	UT VS Gilteritinib	4	0	0.58(0.36, 0.95)*
	UT VS Lestaurtinib	3	19.0	0.98(0.54, 1.78)
	UT VS Midostaurin	3	0	0.75(0.52, 1.08)
	UT VS Sorafenib	3	49.3	1.35(0.81, 2.13)

This table displays pairwise comparative analyses of complete remission (CR) rates for distinct FLT3 inhibitors versus standard therapy (UT). An OR value of less than 1 indicates a more favorable CR outcome in the FLT3 inhibitor group, with statistically significant outcomes marked with. The heterogeneity (%) metric assesses the degree of consistency among the included studies.

### Consistency check

In our network meta-analysis consistency examinations, we evaluated the consistency condition between direct and indirect evidence for two efficacy outcomes, with related results shown in **Table 3**. For the CR outcome, therefore, the consistency test statistic was 42.43, while the inconsistency test statistic was 42.39; this data indicates a high level of consistency between the two models. The heterogeneity index I² reached 1%, hence showing that heterogeneity among the included studies is negligible. For OS, the consistency test statistic was 14.16 and the inconsistency test statistic was 14.17; this further confirms the evidence’s consistency, and the I² value was 0%, which means there is no heterogeneity. Therefore, all above results demonstrate that the consistency test is well fitted with the data, which supports the validity belonging to the network meta-analysis findings.

**Table 3:** Results of consistency and inconsistency test results.

Outcomes	Consistency test	Inconsistency test	I2(%)
CR	42.43	42.39	1
OS	14.16	14.17	0

CR and OS outcomes show good consistency between direct and indirect comparisons. Low I² values reflect acceptable homogeneity of the included studies.

### Complete remission

**Figure 3:**
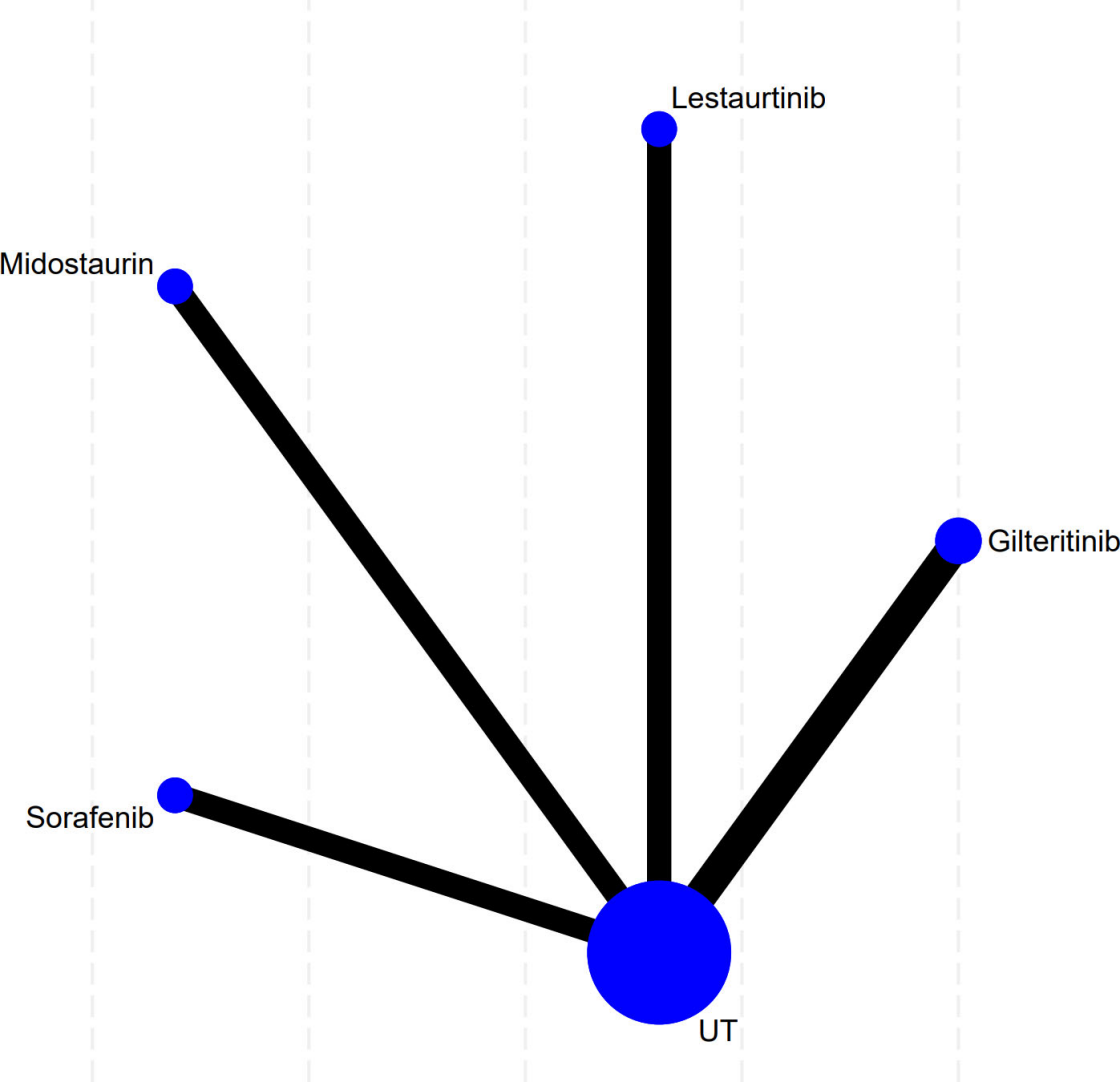
Network evidence plot of complete response. Note: This network plot presents pairwise comparisons of four FLT3 inhibitors versus conventional therapy (UT). Individual nodes correspond to separate treatment arms, and lines link interventions with available direct trial evidence.

This study’s complete remission analysis has included 13 trials. First of all, network diagram results in **Figure 3** demonstrate that evidence network structure of this study takes UT, the control group, as its center, and shows a clear network diagram form. This network has five nodes: four FLT3 inhibitors, namely Gilteritinib, Lestaurtinib, Midostaurin, Sorafenib, and the control group. As what is shown, all FLT3 inhibitors have direct head-to-head comparisons only with UT (black lines). Node size reflects information volume of corresponding treatment plan, with UT node being the biggest. Therefore, this group included the most studies and the largest sample size, and thus it can serve as a strong common reference group for later indirect comparisons. Hence, Gilteritinib has the second-largest sample size, while the other three agents have no obvious differences in their respective sample sizes.

Via **Table 4** of this network meta-analysis, we carried out indirect comparison and quantitative analysis on the relative effectiveness of different FLT3 inhibitors to induce CR in acute myeloid leukemia patients. Therefore, data show that, in comparison with UT, both Gilteritinib (OR = 2.42, 95% CrI: 1.37–4.31, P<0.05) and Midostaurin (OR = 1.81, 95% CrI: 1.17–2.82, P<0.05) could significantly raise CR rates, while no statistically notable difference is seen between these two drugs (OR = 1.34, 95% CrI: 0.85–2.12). Hence, compared with UT, Lestaurtinib (OR = 1.04, 95% CrI: 0.62–1.73) and Sorafenib (OR = 0.72, 95% CrI: 0.49–1.07) did not produce significant CR rate improvement; further analysis found that midostaurin has a significantly higher CR rate than sorafenib (OR = 1.31, 95% CrI: 1.07–1.60).

**Table 4:** Results of the league table for complete response.

OR 95%CrI				
Gilteritinib				
1.69 (0.87, 3.28)	Lestaurtinib			
1.34 (0.85, 2.12)	0.79 (0.45, 1.38)	Midostaurin		
2.42 (1.37, 4.31)*	1.44 (0.75, 2.74)	1.81 (1.17, 2.82)*	Sorafenib	
1.75 (1.16, 2.66)*	1.04 (0.62, 1.73)	1.31 (1.07, 1.6)*	0.72 (0.49, 1.07)	UT

This league table summarizes direct and indirect comparisons of CR rates. Values above 1 in the matrix suggest a superior CR rate for the row treatment versus the column treatment, with *indicating statistical significance. UT serves as the reference for conventional therapy.

**Figure 4:**
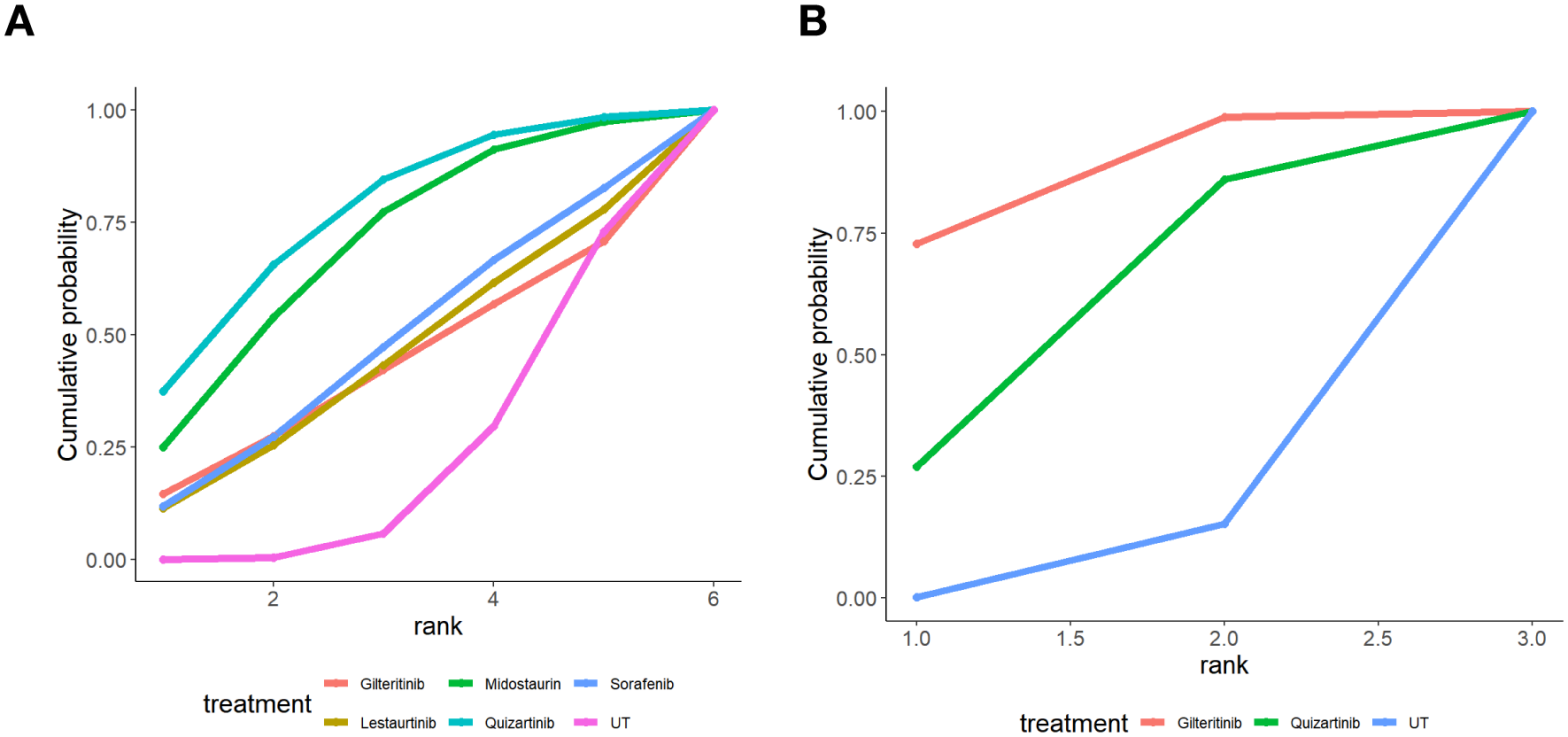
Cumulative probability ranking plot of complete response. **(A)** Newly diagnosed AML: Midostaurin showed the highest probability of being top-ranked for complete remission rates, while sorafenib and UT performed poorly. **(B)** Relapsed/refractory AML: Gilteritinib had the highest ranking probability, indicating superior efficacy in inducing complete remission, whereas UT was the least effective option.

Cumulative probability ranking curves for complete remission (CR) rates, as illustrated in **Figures 4A, B**, together with SUCRA analysis results detailed in **Tables 5–1**, **5-2**, clearly revealed discrepancies in efficacy tiers among various FLT3 inhibitors in patients diagnosed with newly diagnosed or relapsed/refractory (R/R) acute myeloid leukemia (AML). For individuals with newly diagnosed AML (**Figure 4A**), Midostaurin showed the greatest likelihood of achieving high rankings, with a SUCRA value of 86.1%, making it the most suitable agent for improving CR rates; Gilteritinib came next as a secondary option, with a SUCRA score of 66.4%. On the other hand, Sorafenib performed poorly, as its cumulative probability curve only neared 1.0 at the lowest ranking positions, which aligned with its relatively low CR rate of 21.4%. In the R/R AML group (**Figure 4B**), Gilteritinib exhibited remarkable dominance: its steep cumulative probability curve quickly rose to near-peak levels at the highest rank, which corresponded to an 85.9% SUCRA score in **Table 5–2** and the highest CR rate among all treatment strategies assessed. Lestaurtinib was ranked as the second-most effective intervention, while UT was the least effective, indicating that it has limited ability to induce CR in this high-risk patient population. Overall, the results of this network meta-analysis verify that Midostaurin represents the best first-line treatment for patients with newly diagnosed AML, while Gilteritinib surpasses other FLT3 inhibitors in the R/R context when considering CR rates. These findings offer solid evidence to guide clinical decisions regarding personalized treatment options.

**Table 5-1:** SUCRA rankings of CR and OS rates for FLT3 inhibitors in newly diagnosed AML.

Treatment	CR (%)	OS (%)
Gilteritinib	66.4	42.3
Lestaurtinib	23.5	43.8
Midostaurin	86.1	68.9
Sorafenib	21.4	47.1
Quizartinib	NR	76.0

**Table 5-2:** SUCRA rankings of CR and OS rates for FLT3 inhibitors in relapsed/refractory AML.

Treatment	CR (%)	OS (%)
Gilteritinib	85.9	85.8
Lestaurtinib	57.7	NR
Quizartinib	NR	56.4

SUCRA scores quantify the comparative efficacy profiles of individual treatments. NR: not reported; CR: complete remission; OS: overall survival. Newly diagnosed AML: Midostaurin showed the highest SUCRA score for CR (86.1%), while Quizartinib had the highest score for OS (76.0%). Relapsed/refractory AML: Gilteritinib achieved the highest SUCRA scores for both CR (85.9%) and OS (85.8%).

### Overall survival

**Figure 5:**
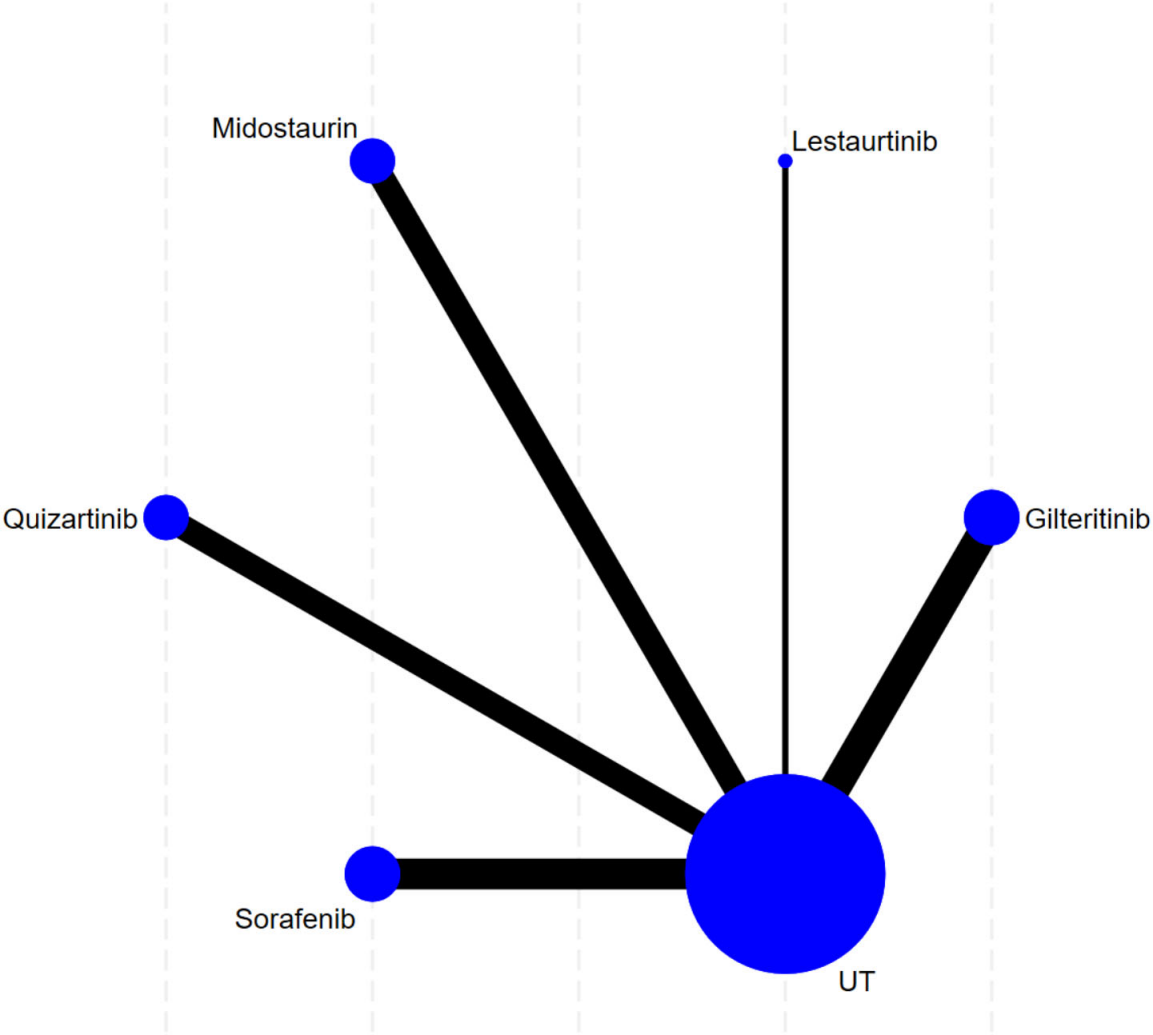
Network evidence plot of overall survival. This figure maps the network architecture for OS outcomes. Each node represents a distinct treatment arm (five FLT3 inhibitors vs. conventional therapy, UT), where node size correlates with the weight of included studies.

A total of 19 studies are included into this OS analysis. **Figure 5** shows the network relations among different FLT3 inhibitors and OS in acute myeloid leukemia patients. The most frequent comparisons are Gilteritinib versus Sorafenib versus control group, with five studies for each, then Quizartinib versus Midostaurin versus control group, with four studies for each. At last, only one study makes comparison between Lestaurtinib and the control group. Concurrently, the maximum node size of UT shows its function as the common reference group, which includes the largest number of studies and sample sizes. Therefore, this robust network structure ensures that we can carry out indirect comparison and ranking of all FLT3 inhibitors’ efficacy through the common control group, thus providing a reliable evidence foundation for subsequent effect size pooling and efficacy analysis.

In this study, we use a network meta-analysis method to make evaluation on the influence that different FLT3 inhibitors exert on survival results of patients having acute myeloid leukemia. As **Table 6** shows, when compare with UT, Gilteritinib HR = 0.70, 95% CrI: 0.49–0.99, P<0.05 and Quizartinib HR = 0.73, 95% CrI: 0.54–0.98, P<0.05 can reduce death risk in significant way, thus bring obvious survival benefits to patients; Midostaurin HR = 0.76, 95% CrI: 0.54–1.08 presents a survival benefit trend but cannot reach statistical significance, while Lestaurtinib HR = 0.90, 95% CrI: 0.55–1.46 and Sorafenib HR = 0.84, 95% CrI: 0.54–1.29 do not show obvious survival advantages. The integrated analysis result reveals that, with survival outcomes as the endpoint, when compare with control group, Gilteritinib and Quizartinib can improve survival prognosis of acute myeloid leukemia patients in significant way. Other FLT3 inhibitors have a survival benefit trend but cannot achieve statistical significance. Therefore, there are no obvious differences in survival benefit between different FLT3 inhibitors, hence this suggests that they may have similar effectiveness in prolonging patient survival time.

**Table 6:** Results of the league table for overall survival.0.

HR 95%CrI					
Gilteritinib					
0.78 (0.43, 1.42)	Lestaurtinib				
0.92 (0.56, 1.51)	1.18 (0.65, 2.15)	Midostaurin			
0.96 (0.61, 1.52)	1.23 (0.69, 2.17)	1.04 (0.66, 1.65)	Quizartinib		
0.84 (0.48, 1.46)	1.07 (0.56, 2.05)	0.91 (0.52, 1.58)	0.87 (0.52, 1.47)	Sorafenib	
0.7 (0.49, 0.99) *	0.9 (0.55, 1.46)	0.76 (0.54, 1.08)	0.73 (0.54, 0.98) *	0.84 (0.54, 1.29)	UT

This matrix presents direct and indirect comparative HR values and 95% CrI for OS outcomes across all treatment groups. Cell values represent the HR of the row intervention versus the column treatment; HR < 1 indicates a favorable survival profile for the row group, with statistically significant outcomes marked with.

**Figure 6:**
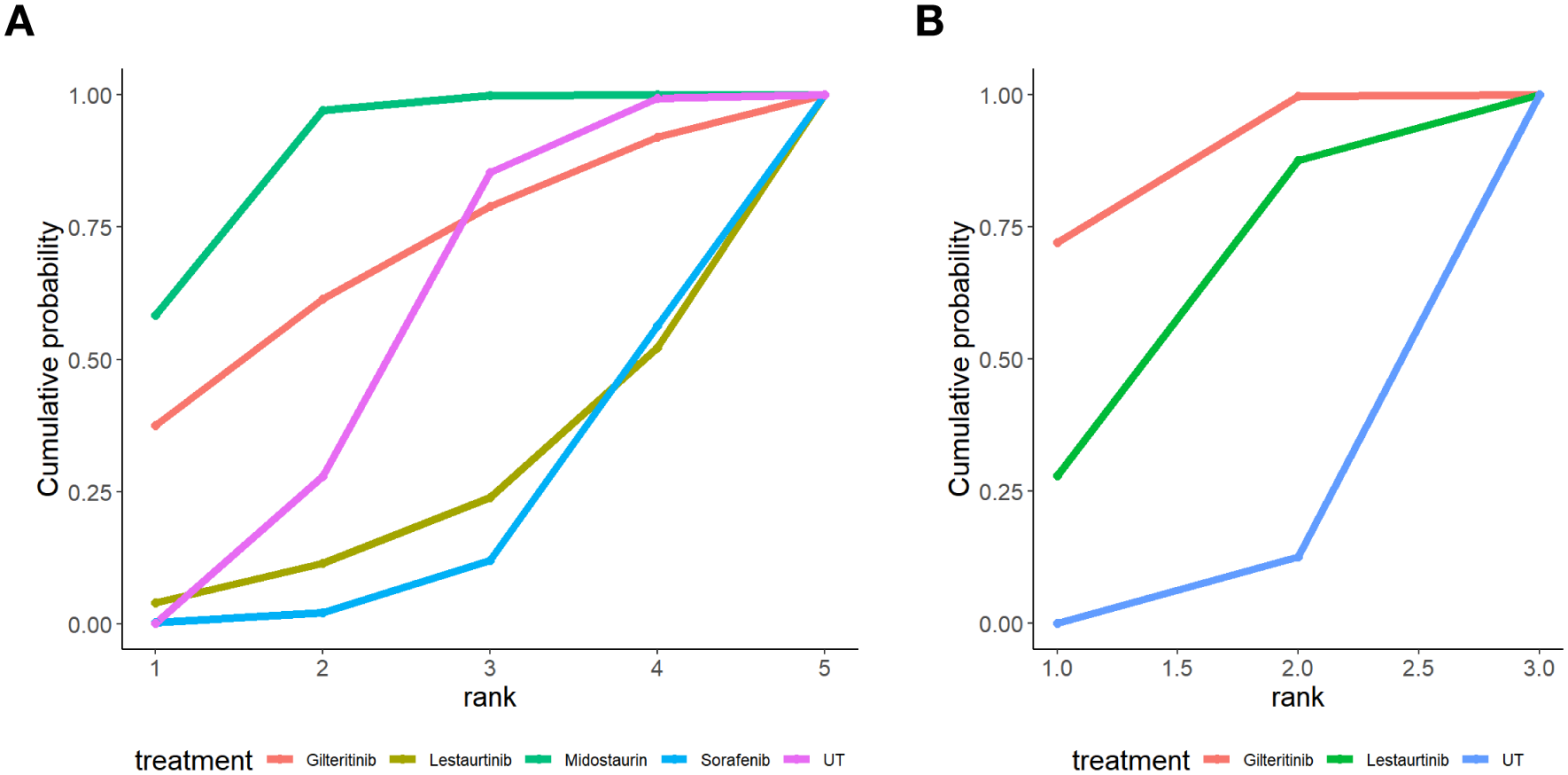
Cumulative probability ranking plot of overall survival. **(A)** Newly diagnosed AML: Quizartinib and Midostaurin displayed the greatest likelihood of securing top rankings for OS, while Lestaurtinib and Sorafenib showed comparatively lower probabilities of being highly ranked. **(B)** Relapsed/refractory AML: Gilteritinib boasted the highest ranking likelihood for OS, reflecting its favorable long-term survival profile, with UT remaining the least effective strategy.

Consistent with the efficacy hierarchy observed for complete remission rates, cumulative probability ranking curves for overall survival (OS) and corresponding SUCRA analyses further delineated differences in long-term benefits of FLT3 inhibitors across subgroups of patients with newly diagnosed and relapsed/refractory (R/R) AML As illustrated by **Figures 6A, B** as well as **Tables 5–1**, **5-2**, the relevant analysis results of this research clearly revealed the variations in OS-related benefits and SUCRA ranking features of FLT3 inhibitors among various AML subgroups. In the newly diagnosed AML cohort (**Figure 6A**, **Tables 5-1**), Quizartinib and Midostaurin exhibited the highest likelihood of achieving top-ranked OS outcomes, with SUCRA values corresponding to survival rates of 76.0% and 68.9%, respectively, indicating substantial long-term survival benefits. Sorafenib and Lestaurtinib showed moderate ranking probabilities, whereas UT presented the lowest probability of attaining favorable survival outcomes, with its cumulative probability approaching 1.0 only at the lowest rank. In the relapsed/refractory (R/R) AML cohort (**Figure 6B**, **Tables 5-2**), Gilteritinib demonstrated absolute superiority in OS outcomes, with its steep cumulative probability curve rapidly rising to near-maximal values at the highest rank, consistent with its high SUCRA score of 85.8% and corresponding survival rate. Quizartinib served as the second-best therapeutic option, while conventional treatment remained the least effective intervention, indicating extremely limited survival benefits of standard therapy in this high-risk population.

Collectively, the findings of the present study confirm that Quizartinib and Midostaurin confer superior OS outcomes in patients with newly diagnosed AML, and identify Gilteritinib as the first-line therapeutic choice for those with relapsed/refractory AML, providing crucial evidence-based support for optimizing clinical decision-making regarding FLT3 inhibitor selection based on disease subtype.

### Adverse events

This research carried out a systematic appraisal of adverse events linked with FLT3 inhibitor treatment for acute myeloid leukemia. As indicated in **Table 7**, majority of adverse events were mild to middle in severity; however, some hematological and biochemical unusual conditions need clinical notice. Among all-grade adverse events, there are neutropenia (RR = 1.57, 95% CI: 1.02–2.43, P = 0.040), anemia (RR = 1.25, 95% CI: 1.10–1.42, P = 0.001), elevated alanine aminotransferase (RR = 1.75, 95% CI: 1.18–2.60, P = 0.006), elevated aspartate aminotransferase (RR = 2.21, 95% CI: 1.69–2.91, P<0.001), fatigue (RR = 1.30, 95% CI: 1.07–1.58, P = 0.009), thrombocytopenia (RR = 1.25, 95% CI: 1.01–1.55, P = 0.041), dyspnea (RR = 3.57, 95% CI: 2.04–6.27, P<0.001), and neutropenia (RR = 1.39, 95% CI: 1.02–1.90, P = 0.037). Among grade 3 or higher adverse events, reduced neutrophil counts (RR = 1.32, 95% CI: 1.02–1.72, P = 0.038), anemia (RR = 1.31, 95% CI: 1.03–1.63, P = 0.002), elevated alanine aminotransferase (RR = 1.36, 95% CI: 1.02–1.83, P = 0.037), elevated aspartate aminotransferase (RR = 2.46, 95% CI: 1.27–4.74, P = 0.007), and neutropenia (RR = 1.39, 95% CI: 1.02–1.90, P = 0.037) are observed. Therefore, hematological toxicity (especially anemia and neutropenia) and hepatic damage (shown by elevated transaminases) are the most noticeable adverse events related to FLT3 inhibitor treatment for acute myeloid leukemia. Hence, this emphasizes that it is necessary to carry out strict monitoring of patients’ complete blood counts and hepatic function indexes in clinical work to guarantee treatment safety.

**Table 7:** Meta-analytic summary of adverse reactions in eligible studies.

Adverse event	Any grade					Grade≥3				
	Study	Heterogeneity		RR (95%CI)	P	Study	Heterogeneity		RR (95%CI)	P
		P	I2(%)				P	I2(%)		
Febrile neutropenia	6	0.049	55.1	1.06(0.85, 1.34)	0.590	9	0.279	18.4	1.03(0.97, 1.09)	0.397
Mucositis	3	0.679	0.0	1.04(0.86,1.26)	0.669	4	0.139	45.5	1.22(0.82, 1.80)	0.330
Fever	4	0.349	9.4	0.98(0.88, 1.10)	0.755	3	0.281	21.3	0.98(0.79, 1.21)	0.818
Hypertension	5	0.039	60.5	1.10(0.60,2.01)	0.675	6	0.167	36	0.97(0.57, 1.65)	0.908
Pneumonia	5	0.692	0.0	1.15(0.98, 1.48)	0.295	8	0.817	0.0	1.14(0.95, 1.38)	0.150
Itching/Pruritus	3	0.981	0.0	0.89(0.62,1.29)	0.535	3	0.25	27.9	0.86(0.16,4.60)	0.863
Neutrophil count decreased	6	0.006	69.6	1.57(1.02,2.43)	0.040*	5	0.157	39.7	1.32( )	0.038*
Sepsis or septic shock	4	0.034	65.3	0.65(0.35,1.20)	0.166	4	0.103	51.5	0.80(0.46,1.39)	0.430
Rash	5	0.996	0.0	1.05(0.91,1.21)	0.512	6	0.056	56.6	1.48(0.76,2.86)	0.249
Abdominal pain	3	0.997	0.0	1.26(0.98,1.62)	0.067	3	0.074	61.6	3.56(0.60,21.26)	0.164
Hypophosphataemia	3	0.467	0.0	1.18(0.75,1.86)	0.465	4	0.654	0.0	1.09(0.67,1.75)	0.732
Hypocalcaemia	3	0.558	0.0	1.12(0.82,1.54)	0.479	4	0	0.523	0.99(0.60,1.63)	0.963
Anemia	8	0.759	0.0	1.25(1.10,1.42)	0.001*	8	0.215	26.8	1.08(1.03,1.13)	0.002*
Pyrexia	4	0.170	40.3	1.13(0.95,1.36)	0.178	5	0.066	54.7	1.31(0.51,3.39)	0.580
Alanine aminotransferase increase	8	0.032	54.5	1.75(1.18,2.60)	0.006*	8	0.69	0.0	1.36(1.02,1.83)	0.037*
Diarrhea	10	0.249	21.0	1.09(0.98,1.21)	0.110	10	0.164	30.6	1.03(0.79,1.34)	0.838
Aspartate aminotransferase increase	6	0.327	13.6	2.21(1.69,2.91)	0.001*	5	0.294	19.0	2.46(1.27,4.74)	0.007*
Hypokalemia	6	0.967	0.0	1.01(0.87,1.17)	0.930	8	0.680	0.0	1.01(0.83,1.23)	0.944
Constipation	7	0.030	56.9	1.15(0.84,1.58)	0.385	6	0.988	0.0	2.70(0.44,16.50)	0.282
Fatigue	7	0.305	16.3	1.30(1.07,1.58)	0.009*	7	0.503	0.0	0.98(0.66,1.46)	0.927
Platelet count decreased	5	0.098	48.9	1.19(0.97,1.45)	0.088	5	0.316	15.4	1.07(0.86,1.32)	0.544
Cough	3	0.050	66.6	1.65(0.84,3.24)	0.142	3	0.956	0.0	1.25(0.13,11.99)	0.844
Thrombocytopenia	6	0.661	0.0	1.25(1.01,1.55)	0.041*	7	0.129	39.4	1.01(0.99,1.04)	0.336
Headache	6	0.007	68.7	1.16(0.82,1.64)	0.396	5	0.568	0.0	1.38(0.41,4.64)	0.608
Peripheral edema	7	0.115	41.3	1.14(0.94,1.38)	0.179	6	0.515	0.0	0.84(0.18,4.03)	0.832
Vomiting	9	0.002	67.7	0.98(0.75,1.29)	0.894	7	0.598	0.0	1.28(0.49,3.33)	0.614
Dyspnea	3	0.703	0.0	3.57(2.04,6.27)	0.001*	4	0.767	0.0	1.30(0.63,2.68)	0.485
Neutropenia	5	0.288	19.9	1.52(1.15,2.02)	0.004*	7	0.001	75.7	1.39(1.02,1.90)	0.037*
Nausea	9	0.019	56.2	0.94(0.77,1.14)	0.534	8	0.466	0.0	0.75(0.49,1.14)	0.179
Infections	6	0.854	0.0	1.17(0.96,1.43)	0.126	5	0.680	0.0	1.02(0.91,1.15)	0.728
Cardiotoxicity or renal insuff	3	0.468	0.0	1.51(0.92,2.47)	0.102	3	0.430	0.0	1.59(0.90,2.81)	0.107
White blood cell count decrease	3	0.010	78.2	2.02(0.50,8.11)	0.321	NR				

This table presents pooled RR estimates and 95% CIs for adverse events of any severity and grade 3 or higher, comparing FLT3 inhibitor regimens to conventional therapy. Asterisks mark statistically significant differences. Study heterogeneity was quantified using the I² metric and corresponding P-value.

CI: confidence interval; NR: Not reported.

### Publication bias

**Figure 7:**
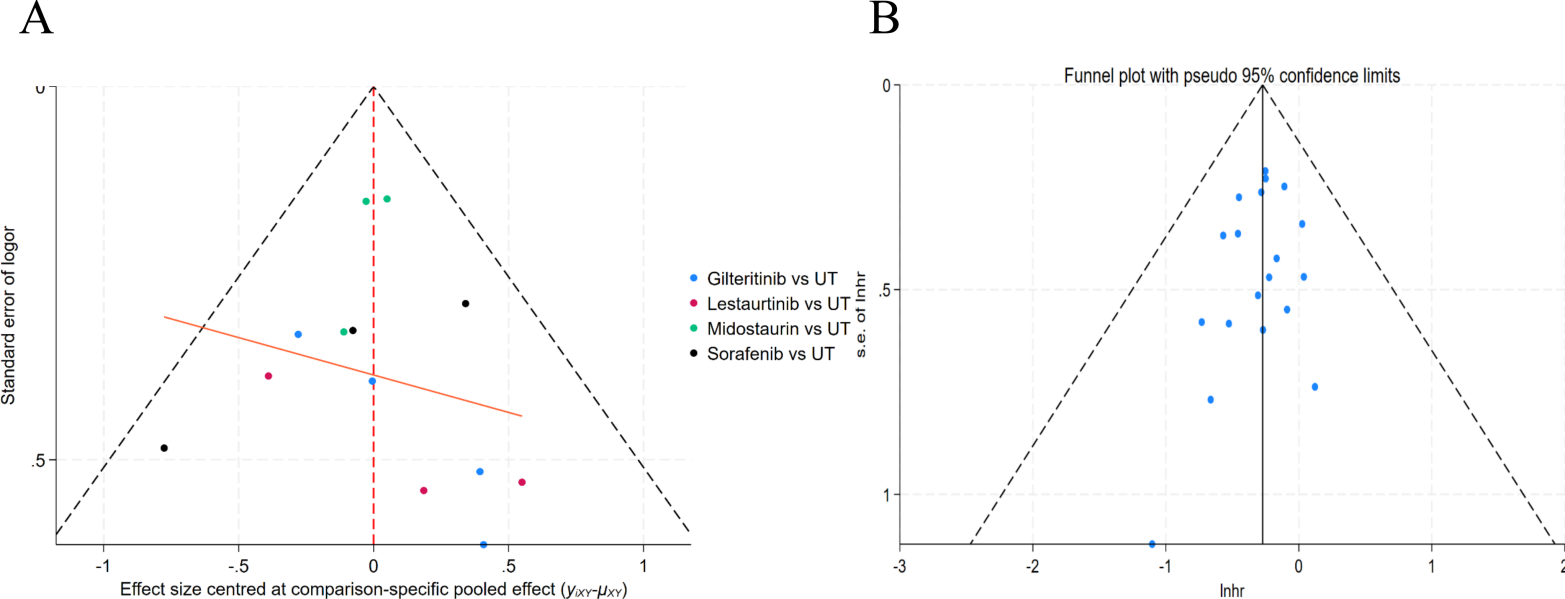
Funnel plots for publication bias assessment of FLT3 inhibitors in acute myeloid leukemia. **(A)** Comparison-corrected funnel plot tailored for network meta-analysis; **(B)** Conventional funnel plot with pseudo 95% confidence bounds. The symmetrical scatter distribution confirms the absence of notable publication bias in the analysis of FLT3 inhibitors for acute myeloid leukemia.

This research used funnel plots to carry out a systematic judgment of publication bias among all included literature. **Figure 7**, which shows funnel plot outcomes for intervention comparisons, proves that effect size data points of gireitinib, letatinib, midoturin, and sorafenib, when set against the UT, show a broadly symmetrical spread around the combined effect size (red dashed line). The whole funnel plot, which takes log-risk ratios (lnHR) as effect measurement tools, further proved that all study data points mostly dropped inside the pseudo 95% confidence interval (black dashed line) and showed fine symmetry around the total combined effect size (black vertical line). Therefore, no obvious one-sided bias or clustering was found; hence, this result further supports the conclusion that this analysis has a low publication bias risk.

## Discussion

FLT3 gene mutations are one of the most widely existing molecular abnormal situations in AML. Among newly diagnosed AML patients, ITD mutation occurrence rate inside FLT3 gene is about 25% (6), and TKD point mutation detection rate is around 7% (35). Therefore, patients who carry this mutation usually have high relapse risk, low cure possibility, and bad disease outcome; hence, FLT3 has become one of the most clinically useful targets in AML targeted therapy. This research used a network meta-analysis method to systematically combine existing clinical evidence, and comprehensively compare the treatment effect and safety of multiple FLT3 inhibitors (Gilteritinib, Quizartinib, Midostaurin, Sorafenib, Lestaurtinib) in AML treatment. It provides a more evidence-based reference basis for AML targeted therapy. The research results of Xinhong Fei (36) et al. show that midotrinib can obviously extend overall survival time of patients. But, this research did not include RCTs of FLT3 inhibitors like gilteritinib and quizartinib to do comparative analysis. It only included one real-world study, which did not report related survival result indexes. Consequently, this study carried out a comprehensive and systematic literature checking work, and included multiple randomized controlled trials that involve FLT3 inhibitors.

In aspect of treatment effect, this study’s discovery shows no difference in therapy response between different FLT3 inhibitors. About the CR endpoint, subgroup analyses were performed in AML patients according to disease status in this study. In patients with newly diagnosed AML, Midostaurin showed the most favorable efficacy, with a SUCRA value of 86.1%. In contrast, Gilteritinib exhibited significant therapeutic advantages in patients with relapsed or refractory AML, with a corresponding SUCRA value of 85.9%. These findings indicate that therapeutic regimens should be tailored to AML patients with different clinical characteristics, providing evidence-based support for individualized targeted therapy in clinical practice. Cumulative probability ranking graphs further verified that they stably took first places in CR rates. As a first-generation FLT3 inhibitor, midostaurin shows significant efficacy in patients with newly diagnosed AML. As a second-generation, high-selectivity FLT3 inhibitor, Gilteritinib has strong blocking function to both FLT3-ITD and FLT3-TKD mutation types. It also can dual-inhibit AXL receptor tyrosine kinase, stop the abnormal downstream FLT3 signal paths, so as to more effectively make leukemia cells die (37). Therefore, Gilteritinib has the biggest possibility to improve key prognosis indexes including CR and OS, thus it is the most hopeful treatment medicine among all FLT3 inhibitors in this analysis. James et al (38) studied Gilteritinib’s pharmacokinetic characteristics, and found it has high target specificity and low off-target influence. At the same time, they raised its oral bioavailability and blood-brain barrier penetration ability, hence it gets better clinical curative effect than other FLT3 inhibitor types (39). Moreover, these results are very consistent with conclusions of Qingyu Xu et al (40), which further proves that Gilteritinib can get best therapy results in clinical environment for relapsed/refractory AML patients with FLT3 gene mutations.

In aspect of survival results, this study also performed subgroup stratified analysis according to patient populations. Data demonstrated that Quizartinib exhibited the most favorable efficacy in patients with newly diagnosed AML, with a SUCRA value of 76%. For relapsed and refractory AML patients, Gilteritinib achieved the best therapeutic effect, with a corresponding SUCRA value of 85.8%. This distribution pattern was highly consistent with the findings for complete remission rate, further confirming the distinct advantage of Gilteritinib in the treatment of relapsed and refractory patients.

As second-generation FLT3 inhibitor, Quizartinib has been proved in clinical studies to obviously lengthen both event-free survival and overall survival in FLT3-ITD-negative AML patients (41). Sorafenib showed no obvious advantage in complete response rates or overall survival rates, a finding closely connected to its limitations as first-generation multi-kinase inhibitor. Therefore, we guess that such drugs may have lower specificity to FLT3, with clear off-target effects, making them less able to continuously block malignant proliferation signals in leukemic cells. Concurrently, through a RCT, Hubert Serve et al (21) reached conclusion that sorafenib, when combined with standard induction and consolidation therapy, brings no benefit to elderly AML patients, and even may produce bad effects. Hence, this is mainly because elderly patients are majority of AML cases, whose reduced physiological tolerance lowers the drug’s pharmacodynamic efficacy and at the same time causes a series of adverse reactions.

In the safety analysis work, this research discovered the core harmful event picture of FLT3 inhibitor treatment for AML. Hematological toxicity and liver damage were the most outstanding and statistically meaningful harmful events, which were seen in all levels and in level 3 or higher events. This matches clinical test data for Gilteritinib, Midostaurin. FLT3 inhibitors inhibit leukemic cell growth and at the same time damage normal hematopoietic stem cell function, which brings about bone marrow suppression. Liver damage mainly has connection with liver metabolism; especially, second-generation inhibitors have higher plasma concentrations that may increase liver metabolism load. At the same time, this research found a very high risk of non-hematological toxicities such as dyspnea and tiredness. Although this phenomenon was reported before, it has not gotten enough attention. Therefore, it emphasizes the need for doctors to give first priority to patients’ subjective feelings and life quality together with laboratory monitoring, and provide timely symptom-related supportive care to raise treatment tolerance degree. Overall, harmful events related to FLT3 inhibitors are mostly mild to middle in seriousness. When compared with clinical benefits, the safety picture shows an acceptable benefit-risk proportion, thus providing big support for their wide clinical use.

The findings of this network meta-analysis offer high-quality evidence-based medical backup for precision therapy of FLT3-mutated AML, thus effectively filling the current blank of comparative evidence related to effectiveness and safety of diverse FLT3 inhibitors in clinical application. FLT3 mutation, as a highly heterogeneous hematological malignancy, is an important adverse prognostic marker in AML (42). Conventional chemotherapy has restricted curative effect on such patients, which is featured by high relapse possibility and short survival time. Therefore, an urgent clinical demand exists for highly effective and safe targeted treatment schemes. The findings of this network meta-analysis offer high-quality evidence-based medical backup for precision therapy of FLT3-mutated AML, thus effectively filling the current blank of comparative evidence related to effectiveness and safety of diverse FLT3 inhibitors in clinical application. FLT3 mutation, as a highly heterogeneous hematological malignancy, is an important adverse prognostic marker in AML. Conventional chemotherapy has restricted curative effect on such patients, which is featured by high relapse possibility and short survival time. Therefore, an urgent clinical demand exists for highly effective and safe targeted treatment schemes.

Although this study has carried out systematic synthesis of existing clinical evidence, certain limitation aspects still exist that demand objective consideration and further polish in follow-up research. Firstly, the evidence network shown here has a lattice structure taking UT as the center; direct head-to-head comparisons among different FLT3 inhibitors are not available here. It mainly depends on indirect comparisons to deduce therapeutic difference between drugs, which may bring about publication bias and heterogeneity, thus possibly weakening evidence reliability. Secondly, baseline differences inside included patient groups, such as genetic mutation subtypes, treatment lines, and comorbidities, were not stratified in a sufficient degree, which has potential to reduce finding accuracy. Finally, this study did not perform deep analysis on the relation among FLT3 inhibitor dosage, treatment cycles, and efficacy or safety outcomes, nor did it take long-term life quality or recurrence rates as endpoint measures. Therefore, the comprehensiveness level of this study’s conclusions needs to be further improved.

Given this study’s limitations and current research progress, future investigations must carry out more strictly designed, sufficiently powered head-to-head clinical trials, so as to directly compare the efficacy and safety situations of different FLT3 inhibitors. Therefore, this can further validate this study’s findings and strengthen the evidence foundation for evidence-based medicine. Secondly, future researches may use stratified analyses; they should combine baseline characteristics like FLT3 mutation subtypes ITD/TKD, treatment lines, age, and performance status to explore personalized treatment plans for different patient groups, thus realizing precision targeted therapy for AML. Finally, long-term follow-up studies should be done, with focus on patient long-term survival, recurrence rates, and life quality. At the same time, deep analysis of the dose-response connection among drug dosage, treatment cycles, efficacy, and safety should be conducted, therefore providing more detailed guidance for standardized clinical practice.

## Conclusions

In summary, this study has carried out comprehensive comparison on the treatment effect and safety of multiple FLT3 inhibitors including Gilteritinib, Quizartinib, and Midostaurin for AML via a network meta-analysis. Therefore, it has verified that FLT3 inhibitors Midostaurin, Gilteritinib and Quizartinib possess obvious superiority in inducing complete disease remission and enhancing patient survival results. Hematological toxicity and hepatic impairment are adverse events that need special clinical attention, hence most adverse reactions are controllable and manageable. Thus, these findings further increase the evidence-based medicine evidence for FLT3 inhibitor therapy in AML, providing important guidance for individual clinical drug prescription, treatment monitoring, and follow-up research. This research has the promise to promote precise targeted therapy in AML and raise clinical results of patients with FLT3-mutated AML.

## Data Availability

The original contributions presented in the study are included in the article/supplementary material. Further inquiries can be directed to the corresponding author.
